# Comparative Susceptibilities of Selected California Chinook Salmon and Steelhead Populations to Isolates of L Genogroup Infectious Hematopoietic Necrosis Virus (IHNV)

**DOI:** 10.3390/ani12131733

**Published:** 2022-07-05

**Authors:** Christin M. Bendorf, Susan C. Yun, Gael Kurath, Ronald P. Hedrick

**Affiliations:** 1 Department of Medicine and Epidemiology, School of Veterinary Medicine, University of California, Davis, CA 95616, USA; cbendorf@csus.edu (C.M.B.); scyun@ucdavis.edu (S.C.Y.); rphedrick@ucdavis.edu (R.P.H.); 2 Department of Biological Sciences, California State University, Sacramento, CA 95819, USA; 3 U.S. Geological Survey, Western Fisheries Research Center, 6505 NE 65th St., Seattle, WA 98115, USA

**Keywords:** IHNV, infectious hematopoietic necrosis virus, Chinook salmon, steelhead trout, experimental challenge, virulence, intraspecies variation

## Abstract

**Simple Summary:**

Chinook salmon in California conservation hatcheries have suffered disease outbreaks and significant mortality due to the fish virus infectious hematopoietic necrosis virus (IHNV) since at least the 1940s. Although steelhead trout in California are also occasionally infected with the same virus they do not typically experience disease. In this study the susceptibility of California Chinook salmon and steelhead trout was defined in controlled experiments that exposed groups of juvenile fish to California IHNV strains (L genogroup). The results confirmed high mortality among Chinook salmon but very low mortality of steelhead trout, despite identical virus exposures. Tests of varying conditions found increasing mortality of Chinook salmon with increasing virus dose, but reduced mortality at higher temperatures or with increased age or size of fish. Among Chinook salmon that survived virus exposure the persistence of virus was detected in one fish 8 months after virus exposure. These findings demonstrate that L genogroup IHNV in California has host specificity for Chinook salmon, and provide an understanding of factors that contribute to disease epidemics in California hatchery Chinook salmon.

**Abstract:**

Salmonid species demonstrate varied susceptibility to the viral pathogen infectious hematopoietic necrosis virus (IHNV). In California conservation hatcheries, juvenile Chinook salmon (*Oncorhynchus tshawytscha)* have experienced disease outbreaks due to L genogroup IHNV since the 1940s, while indigenous steelhead (anadromous *O. mykiss)* appear relatively resistant. To characterize factors contributing to the losses of California salmonid fish due to IHNV, three populations of Chinook salmon and two populations of steelhead native to California watersheds were compared in controlled waterborne challenges with California L genogroup IHNV isolates at viral doses of 10^4^–10^6^ pfu mL^−1^. Chinook salmon fry were moderately to highly susceptible (CPM = 47–87%) when exposed to subgroup LI and LII IHNV. Susceptibility to mortality decreased with increasing age and also with a higher temperature. Mortality for steelhead fry exposed to two IHNV isolates was low (CPM = 1.3–33%). There was little intraspecies variation in susceptibility among populations of Chinook salmon and no differences in virulence between viruses strains. Viral persistence was demonstrated by the isolation of low levels of infectious IHNV from the skin of two juvenile Chinook salmon at 215 d post exposure. The persistence of the virus among Chinook salmon used for stocking into Lake Oroville may be an explanation for the severe epidemics of IHN at the Feather River hatchery in 1998–2002.

## 1. Introduction

Infectious hematopoietic necrosis is a severe rhabdoviral disease of farmed, hatchery and infrequently wild salmonids [1,2,3,4]. In the Pacific Northwest of North America IHNV causes significant mortality in Chinook salmon (*Oncorhynchus tshawytscha*), kokanee (*O. nerka*), sockeye (anadromous *O. nerka*), rainbow trout (*O. mykiss*) and steelhead trout (anadromous *O. mykiss*). Infection with IHNV in juvenile fish can cause lethargic behavior and clinical signs including skin darkening, exophthalmia, hemorrhage or ascites. Pathogenesis involves the degenerative necrosis of the kidney and other hematopoietic tissues. IHN disease may result in mortality up to 100% in young salmonids [3,5], causing significant economic loss for the aquaculture industry and threatening the maintenance of native species propagated for mitigation purposes at hatcheries [1,4]. Control of the disease relies primarily on avoidance of exposure to the virus through the use of virus-free water supplies and surface egg disinfection. Although vaccination procedures are available, costs for the application to the large-scale production of cultured young fish is a concern.

The host range for IHNV includes most salmonids, but the degree of host susceptibility to disease varies among and within species [6,7,8,9,10]. Additional viral, environmental and host factors also contribute to the severity of the disease [11,12]. Production stressors, fish stocking density, co-infections, water quality and temperature significantly impact epidemics, which generally occur in water temperatures from 10–12 °C, with the cessation of mortality usually at temperatures above 15 °C [3,4,11,13]. Mortality resulting from IHNV infection is most severe for young fish, with resistance increasing concurrently with the age and size of the fish [11].

Among IHNV isolates in North America, three genogroups of IHNV, with distinct geographic ranges, are currently recognized: U, M and L for upper, middle and lower regions in the Pacific Northwest of North America [14]. The U genogroup range spans watersheds from Alaska to Oregon, predominantly infecting sockeye and kokanee, but a subgroup of the U genogroup also occurs in Chinook salmon and steelhead in the Columbia River basin of Washington, Oregon, and Idaho [15,16]. The M genogroup is primarily isolated from farmed rainbow trout in southern Idaho and steelhead in hatcheries of the Columbia River basin [16,17,18]. The L genogroup contains all isolates from California and southern coastal Oregon, and two phylogenetic subgroups have been identified: LI is primarily found in Chinook salmon from northern California and Southern Oregon coastal watersheds, and LII is prevalent among Chinook salmon of the inland Sacramento River basin [14,19].

In California, L genogroup IHNV is most commonly encountered among asymptomatic adult Chinook salmon and steelhead returning to spawn at the three largest hatcheries in the Sacramento River basin, the Coleman National Fish Hatchery (CNFH, Anderson), Feather River Hatchery (FRH, Oroville) and Nimbus Hatchery (NH, Gold River). Periodic epidemics due to IHNV have resulted in losses of juvenile Chinook salmon at the CNFH since 1941 [3,4,20,21]. However, no epidemics have occurred there since the initiation of the ozone treatment of the water supply in 1999. The NH has experienced sporadic epidemics of IHNV in Chinook salmon fry, and the FRH was plagued with IHNV epidemics in young Chinook salmon in the late 1990’s and early 2000’s. Although most disease occurs in Chinook salmon, in 2001, FRH reported the first IHNV outbreak in steelhead since its inception in 1969, with the loss of 39% of the steelhead produced that year. The outbreak of IHNV in steelhead at the FRH in 2001 was of particular concern, with respect to the potential for virus evolution similar to that suggested for the M genogroup, which is hypothesized to have arisen by a species jump from sockeye salmon into rainbow trout [14,18,22].

An investigation into the possible causes of the 1998–2002 epidemics at the FRH using phylogenetics and virulence studies provided evidence that the virus associated with the epidemics at the FRH was genetically consistent with and no more virulent for Chinook salmon and rainbow trout than historical isolates in the water system [19,23]. Instead, recurring epidemics at the FRH were suspected to have resulted from the higher concentrations and prevalence of the virus in the water system due to the high-density stocking of IHNV-susceptible fish and potential IHNV carriers into Lake Oroville above the hatchery water supply. A second possible explanation for the epidemics could be changes or variation in the virus susceptibility of strains of FRH fall-run Chinook salmon or steelhead present in the hatchery. Here we investigate both of these hypotheses by conducting controlled experimental challenges of California salmonids with California isolates of IHNV. The objectives of this study were to: (1) determine if susceptibility to L genogroup IHNV differs among selected Chinook salmon and steelhead populations resident in California watersheds; (2) evaluate the effect of virus concentration, water temperature and host age on mortality as factors contributing to disease that may explain the preponderance of epidemics at the FRH; and (3) examine the persistence of IHNV in California Chinook salmon and steelhead.

In previous studies, viral virulence and variation in host susceptibility have been characterized for IHNV from the U and M genogroups in hosts from their respective northern geographic ranges [9,10,11,24,25,26]. In these studies, L genogroup virus strains have sometimes been included, but the virulence of LI and LII subgroup IHNV and variation in the susceptibility of the natural hosts of L genogroup virus, Chinook salmon and steelhead of California, has not previously been characterized. Our study comprises a thorough experimental investigation of California salmonid interactions with L genogroup IHNV, and the testing of multiple populations of each host species complements recent studies of intraspecies variation in IHNV susceptibility among more northern populations of Chinook salmon conducted by Hernandez et al. [25,26].

## 2. Materials and Methods

### 2.1. Fish Populations

Fish from three populations of fall-run Chinook salmon and two populations of steelhead trout native to California watersheds were used in experimental virus challenge studies (Table 1).

Fish were received as eggs, hatched and reared with a biodiet ration (BioProducts, Inc., Astoria, OR, USA) in 36-L aquaria supplied with 12 °C pathogen-free well water at a rate of 0.2 L min^−1^. The Feather River (FRH) and Nimbus (NH) Chinook salmon populations are from Sacramento River basin hatcheries that have experienced IHN epidemics and are in the geographic range of the LII subgroup of IHNV. Both of these populations are considered to be genetically homogeneous as determined by allozyme, mitochondrial DNA and microsatellite loci analyses [27,28,29,30,31,32,33,34,35]. The Irongate population (IGH) of Chinook salmon is from a northern California coastal watershed in the geographic range of the LI subgroup of IHNV, but Irongate Hatchery has no history of IHN epidemics. This population is considered genetically heterogeneous [27,28,29,33]. The FRH and Coleman National Fish Hatchery (CNFH) steelhead populations are both from Sacramento River basin hatcheries within the LII geographic range. These populations are genetically homogenous but distinct [36]. The Coleman National fish hatchery has no record of steelhead epidemics despite a history of epidemics in Chinook salmon, and the CNFH steelhead have been reported to be refractory to IHNV infection [37].

### 2.2. Virus

IHN viruses are members of the species *Novirhabdovirus salmonid*, genus *Novirhabdovirus*, family *Rhabdoviridae.* Five IHNV isolates used in experimental challenge studies are described in Table 2. 

Complete isolate designations from fish health agencies or names presented in Kelley et al. [19] are shown, and abbreviated names of virus isolates will be used hereafter. Isolate FR04 was obtained from the California Department of Fish and Game (CDFG); RR98 from the Oregon Department of Fish and Wildlife (ODFW); and CL02, FR92, and FR69 were from the U.S. Fish and Wildlife Service. Four of the isolates had been passaged either 2 or 3 times prior to use in our studies, but passage number was unknown for CL02. Virus isolates were grown in the CHSE-214 (ATCC CRL 1681) cell line established from Chinook salmon [38] or the epithelioma papulosum cyprini (EPC) line [39,40] using standard methods [41]. Viruses were stored at −80 °C as frozen aliquots of cell culture supernatant. Both cell lines were propagated according to Arkush et al. [42] in minimum essential medium (MEM) buffered to pH 7.8 with a reduction from 7.5% fetal bovine serum for routine cell culture to 2% (MEM-2) following virus infection.

### 2.3. Experimental Studies of Fish Susceptibility to Mortality

Three virus exposure experiments were conducted to determine the kinetics and final levels of mortality after the exposure of various fish populations to selected IHNV strains under varying conditions (Table 3).

Fish were challenged by exposure to IHNV isolates at relative viral concentrations of approximately 10^4^ (low), 10^5^ (intermediate) or 10^6^ (high) pfu mL^−1^, and actual virus exposure doses were determined directly from the virus suspensions on the day of each challenge [23]. The determination of viral titer using plaque assays buffered with HEPES (MEM-2H) followed the procedure adapted from Arkush et al. [42], as described by Bendorf et al. [23]. For Experiment 1, three populations of Chinook salmon fry were exposed to two IHNV isolates at 12 °C. Fry of the FRH, NH and IGH were grown to an age of 588 degree days (dd, in °C) and weighed an average of 0.54, 0.57 and 0.55 g, respectively, at the time of virus exposure. They were challenged by exposure to IHNV isolates FR04 and RR98 at three relative viral concentrations. Virus concentrations determined on the day of challenge (actual challenge doses) were 4.6 × 10^4^, 4.6 × 10^5^ and 4.6 × 10^6^ pfu mL^−1^ for FR04 and 5.7 × 10^4^, 5.7 × 10^5^ and 5.7 × 10^6^ pfu mL^−1^ for RR98. In Experiment 2, Chinook salmon fingerlings from FRH and IGH were 1260 dd in age and 2.2 g in average weight at the time of challenge. They were exposed to intermediate and high relative concentrations of the FR04 IHNV isolate at three water temperatures, 10 °C, 12 °C and 14 °C, to simulate temperature variation consistent with temperatures at the FRH and IGH. Actual exposure doses of FR04 were 7.0 × 10^5^ (intermediate) and 7.0 × 10^6^ pfu mL^−1^ (high).

In Experiment 3, steelhead fry from FRH and CNFH were 714 dd in age and weighed approximately 0.68 and 0.67 g, respectively. They were exposed to three doses of IHNV isolates FR04 and CL02. Actual challenge doses were 1.2 × 10^4^, 1.2 × 10^5^ and 1.2 × 10^6^ pfu mL^−1^ for FR04 and 2.0 × 10^4^, 2.0 × 10^5^ and 2.0 × 10^6^ pfu mL^−1^ for CL02.

For each experiment, duplicate groups of 30 fish were challenged by static immersion in water containing dilutions of cell culture medium with each IHNV isolate at each challenge dose. Duplicate control groups of 30 fish received only MEM-2 without virus. Exposures were conducted by placing fish into 0.5 L (for Chinook salmon and steelhead fry, Experiments 1 and 3) or 1.0 L (Chinook salmon fingerlings, Experiment 2) of aerated well water with or without virus in 1.7 L plastic containers immersed in flowing well water, to maintain temperature. After 1 h, the fish were transferred to 15 L aquaria maintained in an aerated flow-through system supplied with chilled well water. Cumulative percent mortality (CPM) was recorded daily over a 21 d period post-virus exposure. At 21 d following the initial virus exposure, all remaining surviving fish were euthanized in a 500 mg/L benzocaine solution.

### 2.4. Temperature

All virus exposures were conducted at a water temperature of 12 °C, except Chinook salmon fingerlings in Experiment 2, which were tested at three water temperatures (10 °C, 12 °C and 14 °C). Fish stocks originally at 12 °C were adjusted gradually (0.5 °C) daily to reach the desired water temperature 3 days prior to virus exposure.

### 2.5. Viral Persistence Studies

Longer experiments were conducted to examine the potential persistence of the virus beyond the standard 21 d duration of the mortality studies. Chinook salmon fingerlings originating from NH (5.1 g) and IGH (4.2 g) and 1554 dd in age were exposed to a low dose of FR04 virus concentration (10^4^ pfu mL^−1^). FRH steelhead fry were exposed to a low dose of FR04 or CL02 in mortality Experiment 3 as described above. Lastly, FRH steelhead fingerlings (1.1 g) at 834 dd in age were exposed to a high dose of FR92 or FR69 virus, relative concentration (10^6^ pfu mL^−1^). After virus exposure, fish were held and sampled at defined time points. For Chinook salmon, at 120 d post exposure (dpe), 4–6 gill filaments, kidney and spleen tissues were collected as separate samples from 30 fish in each Chinook salmon exposure group and respective control groups (a total of 60 IHNV-exposed Chinook salmon and 60 unexposed control fish). A second and final examination of 32 Chinook salmon (28.9 g) occurred at 215–216 dpe with the same tissues sampled as at 120 dpe but also including a portion of the abdominal skin. Skin samples of approximately 0.05 g were taken as slices near the lateral line between the dorsal and pelvic fins and represented an area of approximately 4 × 20 mm. A total of 163 steelhead (22.7 g) were sampled as described above, including skin samples, at 227–248 dpe. Control unexposed fish sampled at the later collection dates included 30 Chinook salmon and 45 steelhead. Samples were processed fresh on the day of collection, using methods described above for determining presence and concentrations of viable IHNV by plaque assay.

### 2.6. Statistical Analyses

The correlation between parameters of virus, virus concentration (exposure dose), water temperature and fish stock were analyzed using the Glimmix model statistical program (SAS Institute Inc., Cary, NC, USA). Mortality curves were estimated with the Kaplan–Meier method and compared with a log-rank test with adjustment for sample size by Tukey-Kramer. Data that presented with a *p* value of <0.05 were considered statistically significant and thus would implicate a strong correlation between the parameters examined.

## 3. Results

### 3.1. Chinook Salmon Fry Susceptibility at 12 °C

**Figure 1:**
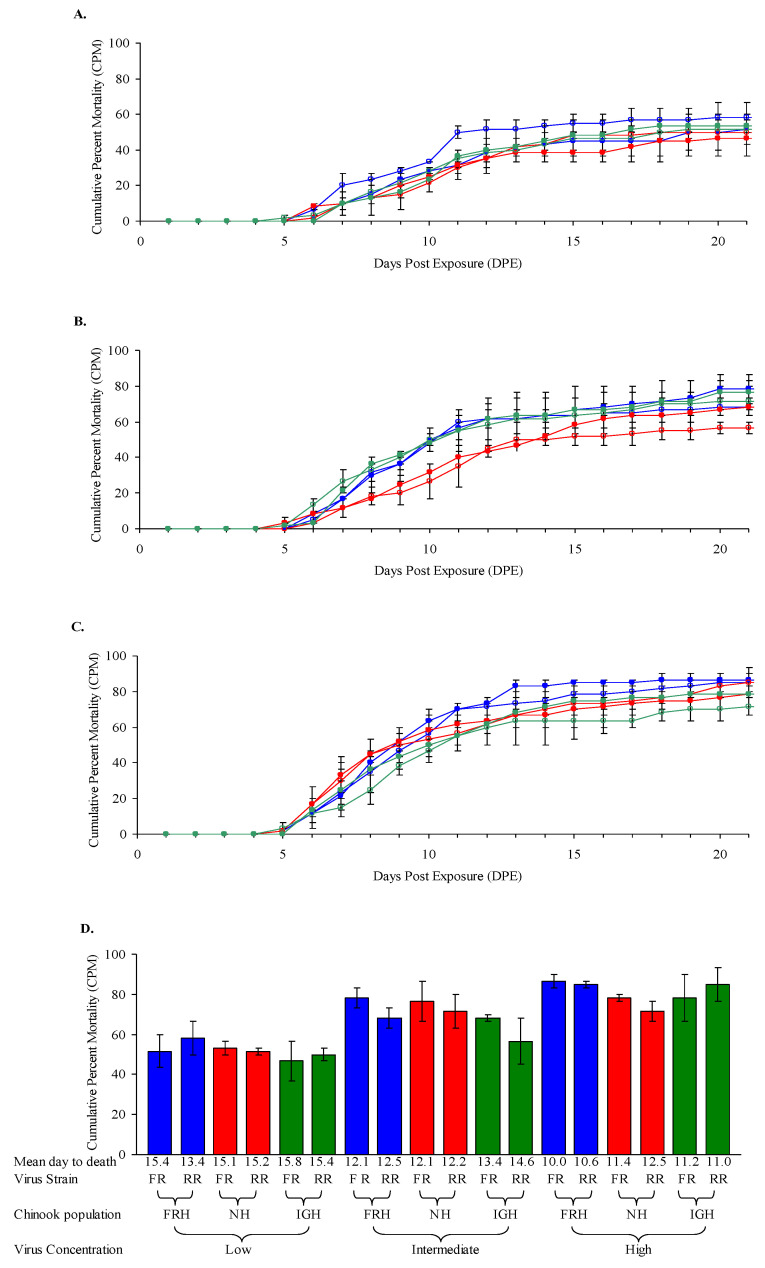
**Mortality of Chinook salmon fry exposed to IHNV at 12 °C.** Average daily cumulative percent mortality (CPM) is shown for duplicate groups of 30 Feather River Hatchery (FRH) Chinook salmon (blue), Nimbus Hatchery (NH) (red) and Iron Gate Hatchery (IGH) Chinook salmon (green) fry (0.54–0.57 g) after waterborne exposure to (**A**) low, (**B**) intermediate and (**C**) high doses of infectious hematopoietic necrosis virus subgroup LII isolate FR04 (filled symbols) or subgroup LI isolate RR98 (open symbols) at a water temperature of 12 °C. Actual exposure doses determined at the time of challenge were: RR98, 5.7 × 10^4^, 5.7 × 10^5^, and 5.7 × 10^6^ pfu mL^−1^; and FR04, 4.6 × 10^4^, 4.6 × 10^5^, and 4.6 × 10^6^ pfu mL^−1^. (**D**) Bars show average CPM (±SE) in duplicate treatment groups.

Chinook salmon fry (average weight 0.54–0.57 g) from three different populations demonstrated a moderate to high level of susceptibility to two L genogroup IHNV isolates, with cumulative percent mortality (CPM) ranging from 47–87% depending on virus dose (Figure 1).

There were no significant differences in mortality rates (mean day to death, MDD) or CPM between Chinook salmon fry of different hatchery origins exposed to either the RR98 (LI) or FR04 (LII) genotypes of IHNV at each exposure dose. Significant differences in mortality were observed between Chinook salmon fry exposed to different concentrations of IHNV (*p* value < 0.0001) for both the RR98 and FR04 IHNV isolates, with cumulative percent mortality (CPM) increasing in correlation with greater virus doses (Figure 1). Although just below significance (*p* = 0.0562), the NH Chinook salmon appeared slightly more resistant than the FRH fish at the highest virus dose based upon CPM and MDD = 11.4–12.5 and 10.0–10.6, respectively (Figure 1C,D). No mortality was observed over the 21 d period among unexposed control groups of Chinook salmon fry.

### 3.2. Chinook Salmon Fingerling Susceptibility at Three Temperatures

**Figure 2:**
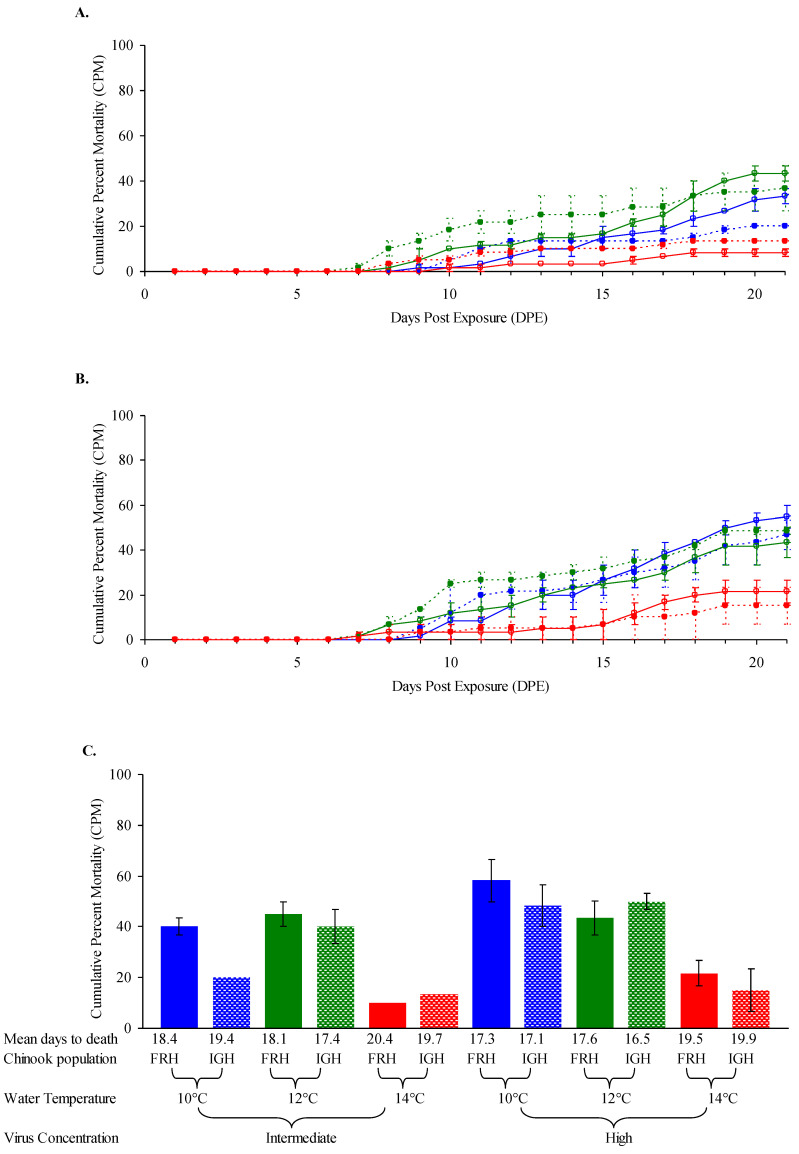
**Mortality of Chinook salmon fingerlings exposed to IHNV at three temperatures.** Average daily cumulative percent mortality (CPM) for duplicate groups of 30 Feather River Hatchery (FRH) Chinook salmon (solid line) and Iron Gate Hatchery (IGH) Chinook salmon (broken line) fingerlings (ave. 2.2 g) after waterborne exposure to (**A**) intermediate and (**B**) high doses of infectious hematopoietic necrosis virus subgroup LII isolate FR04 at water temperatures of 10 °C (blue), 12 °C (green) and 14 °C (red). Actual exposure doses determined on the day of challenge were 7.0 × 10^5^ and 7.0 × 10^6^ pfu mL^−1^. (**C**) Bars show average CPM (±SE) in duplicate treatment groups. Absence of SE bars indicates that duplicate groups had identical CPMs.

Two populations of Chinook salmon fingerlings (ave. 2.2 g) exposed to intermediate and high doses of IHNV isolate FR04 at 10, 12 or 14 °C had CPM that ranged from 10 to 58% (Figure 2). The CPM did not differ significantly between fish populations but varied significantly depending on virus dose (*p* = 0.0282) and with water temperature (*p* < 0.0001). Moderate mortality (40–58%) following exposure to IHNV was observed among Chinook salmon fingerlings originating from both the FRH or IGH at water temperatures of 12 °C and 10 °C, with the exception of significantly (*p* = 0.0282) lower mortality (20%) for IGH at the intermediate virus dose at 10 °C (Figure 2). Mortality for both fish stocks significantly declined (*p* < 0.0001) at a water temperature of 14 °C (CPM = 10–22%). No mortality was observed in unexposed control groups of Chinook salmon fingerlings over the 21 day period.

### 3.3. Steelhead Fry Susceptibility at 12 °C

**Figure 3:**
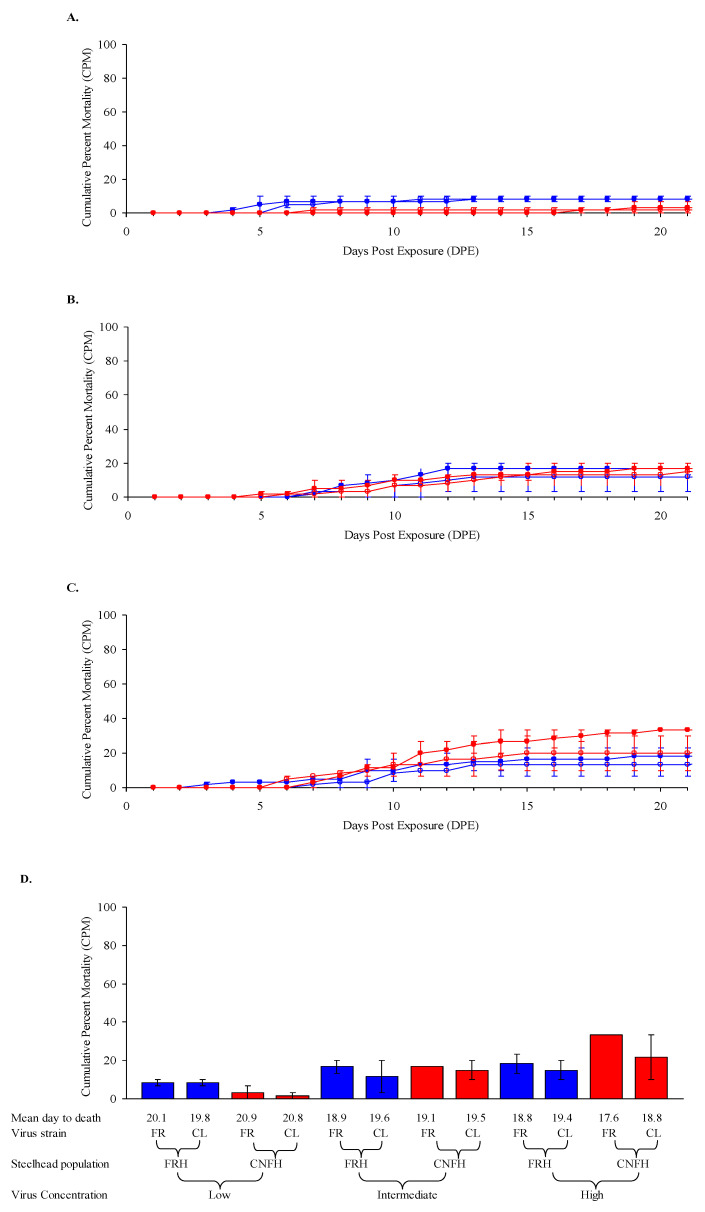
**Mortality of steelhead fry exposed to IHNV at 12 °C.** Average daily cumulative percent mortality (CPM) for duplicate groups of 30 Feather River Hatchery (FRH) (blue) and Coleman National Fish Hatchery (CNFH) steelhead (red) fry after waterborne exposure to (**A**) low, (**B**) intermediate and (**C**) high doses of infectious hematopoietic necrosis virus subgroup LII isolates FR04 (filled symbols) and CL02 (open symbols) at a water temperature of 12 °C. Actual exposure doses determined on the day of challenge were: FRH, 1.2 × 10^4^, 1.2 × 10^5^, and 1.2 × 10^6^ pfu mL^−1^; and CNFH, 2.0 × 10^4^, 2.0 × 10^5^, and 2.0 × 10^6^ pfu mL^−1^. (**D**) Bars show average CPM (±SE) in duplicate treatment groups. Absence of SE bars indicates that duplicate groups had identical CPMs.

Overall, two populations of steelhead fry (0.67–0.68 g) showed a low susceptibility to IHNV, with CPM ranging from 1.7 to 33% after low-, intermediate- or high-dose exposures. There was no significant difference in the susceptibility of either population of steelhead fry (FRH or CNFH) following exposures to LII IHNV isolates FR04 or CL02 at the same virus dose (Figure 3). Significant differences in mortality (*p* value < 0.0001) were observed between fish exposed to different concentrations of both IHNV isolates (Figure 3). Mortality among both steelhead stocks exposed to the lowest concentration of the CL02 isolate was not significantly different from the control group (Figure 3). A trend was observed for a more narrow mortality range (8.3–18.3%) of steelhead from the FRH compared to that experienced by fish from the CNFH (1.7–33%) in response to increasing doses of either virus. A CPM of 1.7% and 3.3% was observed in the unexposed control groups for the FRH and CNFH steelhead stocks, respectively, and a fresh mount examination of the spleen from one of the dead fish revealed long slender rods suggestive of *Flavobacterium psychrophilum*. Virus was not isolated from any control fish or from fish dying in the low-dose exposures but was isolated from all fish dying in the intermediate and high virus treatment groups.

### 3.4. Viral Persistence

The ability of IHNV to persist in Chinook salmon and steelhead was assessed by plaque assay for viable virus in individual fish tissue samples collected approximately 4 or 7 months after exposure to virus (Table 4). There was no virus detected upon examination of the kidney, spleen or gills of Chinook salmon from NH and IGH 120 d after exposure to a low dose of FR04 IHNV. In contrast, when skin tissues were included in the sampling, virus was detected in the skin of 2 of 32 asymptomatic Chinook salmon examined at 215–216 dpe. Concentrations of virus present in the skin were 2.5 × 10^2^ to 1.5 × 10^3^ pfu g^−1^ which was at or near the lower limits of detection of 2.5 × 10^2^ pfu g^−1^ for the plaque assay employed. Virus was not detected in any of the 163 FRH steelhead sampled at 227–248 dpe (Table 4). No virus was detected from control fish of either species, at any time point.

## 4. Discussion

The current study examined susceptibility of three Chinook salmon and two steelhead populations from California hatcheries to L genogroup IHNV isolates. Fish and virus populations were from the geographic ranges of both subgroup LI and LII IHNV in California and included watershed specific IHNV isolates to investigate potential factors contributing to severe epidemics among Chinook salmon and juvenile steelhead at the FRH in 1998 and 2000–2002. In the current study, the moderate to high susceptibility of three native California Chinook salmon stocks exposed to L genogroup IHNV at approximately 0.5 g in size was consistent with the high incidence of IHNV epidemics observed in juvenile Chinook salmon at California hatcheries where L genogroup IHNV is endemic. In accord with Bendorf et al. [23], who examined historical LI and LII isolates from epidemics at FRH, experimental challenges in the current study demonstrated no significant differences in the virulence of LI and LII virus isolates and no intraspecies variation in susceptibility for juvenile Chinook salmon from three hatchery origins (FRH, IGH, NH). This suggests that virulence for selected populations of Chinook salmon from California has not changed despite the genetic divergence of the IHNV into the LI and LII subgroups [19]. The significant increase in CPM (*p* value < 0.0001) observed in correlation with greater virus dose has been described in most challenge studies with IHNV, e.g., [10,11,12,25].

In comparison with a prior report that very young Chinook fry (10 days post-hatch) had high initial susceptibility to IHNV (CPM = 80–100%) but developed complete resistance by 30 days post-hatch [21], and our observation of susceptibility of older Chinook salmon is likely due to the higher virus concentrations used in our study (10^2−4^ fold greater). The mortality (CPM = 47–87%) observed for the three stocks of Chinook salmon fry in our study is in the range (CPM = 47–72%) reported for similar aged NH Chinook salmon (0.56 g) to equivalent concentrations of seven L genogroup isolates at 12 °C [23]. Moreover, the mortality in our study is similar to the CPM of 40–93% reported for Chinook salmon fry (0.6–0.9 g) from Oregon and California, USA, following exposures to intermediate and high concentrations of a presumed L genogroup IHNV [8]. In recent investigations of Washington and Oregon Chinook salmon populations (1 g) exposed to an LII subgroup IHNV from FRH at concentrations comparable to those used in our study (~10^5^ pfu mL^−1^), mortality ranged from 17–49% [25,26]. This is lower than the mortality we observed for 0.5 g fry but similar to the mortality we observed in fingerling Chinook salmon at 2.2 g. In contrast to these studies, Chinook salmon stocks (0.5 g) native to the states of Alaska and Washington had low mortality (CPM = 1–4% and 2–27%, respectively) after exposure to low and intermediate doses of a presumed L genogroup isolate from CNFH [43]. Further studies are needed to determine whether more northern stocks of Chinook salmon are potentially more resistant than those in the southern range of IHNV. However, in general, Chinook salmon fry originating within the L genogroup range [14] are consistently moderately to highly susceptible to both LI and LII IHNV in 10–12 °C water at virus concentrations at or above 10^4^ pfu mL^−1^. Among the selected groups of Chinook salmon from California examined here, virus concentration, not isolate nor fish stock origin, significantly impacted mortality to L genogroup IHNV. These finding suggest that a key element in the epidemics observed at the FRH are increased concentrations of virus in the water supply, rather than host stock or watershed-specific virulence of the virus isolates.

In our current study, the susceptibility of FRH and IGH Chinook salmon fingerlings exposed to two concentrations of an LII IHNV isolate was used to evaluate the effects of host age and water temperature (10 °C, 12 °C and 14 °C) on viral virulence. Chinook salmon fingerlings, aged 1260 dd, from the FRH and IGH demonstrated an increased resistance (CPM = 40–50%), compared to their younger counterparts (CPM = 68–87%), following exposure to intermediate and high doses of a LII IHNV isolate at a water temperature of 12 °C. Increased resistance concurrent with the age and size of the host has been previously reported for several species of salmonids including Chinook salmon and is thought to be due at least in part to greater immune competence in older fish [3,4,6,8,25,44,45]. Chinook salmon fingerlings in our study were also significantly more resistant to IHNV at a water temperature of 14 °C. This finding is consistent with prior reports of the protective effect of higher water temperature, which is hypothesized to enhance the fish immune response, as well as physiology and metabolism [3,4,11,13,46]. Our finding supports the potential and continued application of elevated water temperature as a means to control IHNV at California hatcheries [13,47]. This form of control, however, should be approached cautiously since decreased sensitivity to elevated water temperatures for recent isolates from the CNFH has been reported [47]. There was no difference in susceptibility between stocks of Chinook salmon fingerlings exposed to virus at 12 °C or to the high virus dose at 10 °C. However, when challenged at an intermediate dose of IHNV at a water temperature of 10 °C, the IGH stock of Klamath Basin Chinook salmon was more resistant to IHNV-induced mortality than Central Valley Chinook salmon originating from the FRH. Water temperatures at the FRH remain between 10–12 °C for prolonged periods during the rearing of Chinook salmon (March to mid-May), an effect due to the establishment of the Oroville dam [21]. This may provide an extended optimal temperature range for the onset of IHNV epidemics, which is suggested by the mortality rates observed for FRH Chinook salmon exposed to IHNV at water temperatures of 10 °C and 12 °C in our experimental trials. In contrast, water temperatures at the IGH are consistently cooler through the early rearing of Chinook salmon, a factor that might contribute to a diminished effect of IHNV. Further investigation into the difference in resistance observed for IGH and FRH Chinook salmon at 10 °C would be necessary to determine if the higher susceptibility of FRH Chinook indicates a more IHNV susceptible Central Valley stock.

Among steelhead fry (~0.7 g) from the FRH and CNFH, even at virus concentrations considered environmentally high [47], both LII isolates of IHNV induced considerably less mortality (CPM = 1.3–33%) than that observed among Chinook salmon fry and fingerlings. This low susceptibility to LII IHNV is likely a major reason for the general lack of epidemics for Central Valley steelhead and low prevalence of IHNV in asymptomatic adults returning to both hatchery watersheds. The similar response of both steelhead stocks to IHNV infection was anticipated given the extreme low level of genetic variability among the viral genotypes present in the two water systems [19] and the genetic homogeneity of Central Valley steelhead [36]. The experimental challenges in our study clearly demonstrate the low susceptibility of Central Valley steelhead fry, with the development of complete resistance of fingerlings by 4.1 months in age (C.M.B., unpublished data). Very young rainbow trout and steelhead fry (ca. 0.2 g) originating from stocks outside of California have been reported as highly susceptible (80% CPM) to laboratory exposures with L genogroup IHNV [48]. This initial susceptibility for rainbow trout and steelhead then decreased concurrent with host size increases (2.6–2.8 g for rainbow trout, 0.4 g for steelhead), with the development of complete resistance to mortality a few months post hatch following experimental exposures to IHNV [7,8,9,23,48]. Two prior studies with steelhead and rainbow trout of California origin reported an even more rapid onset of complete resistance to IHNV (presumed L genogroup)-induced mortality [21,37]. The higher virus exposure doses (10–100 fold greater) in our study likely explain the more delayed onset of resistance. The presence of *F. psychrophilum* in the steelhead in our trial may have contributed to an increased susceptibility to IHNV as co-infections with these two agents are commonly involved in mortality during rainbow trout production [49]. Nonetheless, there is a window of less than 4.1 months, likely an overestimate based on environmentally relevant concentrations of IHNV, for which California steelhead demonstrate low but significant susceptibility to L genogroup IHNV at 12 °C.

Persistence of IHNV in populations of Chinook salmon or rainbow trout in Lake Oroville upstream of the water source for the FRH has been suggested as a potential source of virus for the epidemics at the FRH in 1998 and 2000–2002 [23]. Typically virus has been isolated from salmonids shortly after infection, but it has also been hypothesized that IHNV may persist at an undetectable level in a life-long carrier state until fish come to maturity at spawning [4,21,50,51,52,53]. That persistence of the virus is possible has been proposed as Drolet et al. [54] demonstrated the presence of defective interfering particles 1–2 years post viral exposure in kidney and liver tissues from rainbow trout. Additionally, virus-exposed rainbow trout and Chinook salmon have been shown to remain seropositive for IHNV antibodies up to 18 months post exposure to IHNV [55,56,57]. Mortality among Chinook salmon and recovery of IHNV at 179 dpe has been demonstrated in experimental challenges in seawater [57]. More typically rainbow trout and Chinook salmon that survive exposures to IHNV test negative for IHNV in samples from hematopoietic tissues when examined at or beyond 60 dpe [44,47,54,57,58]. The detection of infectious IHNV in our study at 215 dpe from the skin of 2 of 32 healthy appearing yearling Chinook is supporting evidence that this may be a site of viral persistence [59]. The lack of isolation of infectious virus from steelhead surviving exposures to IHNV suggest that virus may be more effectively cleared than in Chinook salmon, but this is not conclusive due to the low frequency of detection in Chinook salmon. Still, it is tempting to suspect that the ability for the virus to persist in Chinook salmon has been an advantage for the L genogroup virus in this salmonid species. In contrast, the greater resistance to L genogroup IHNV observed in steelhead (including lack of virus persistence) explains the paucity of epidemics in this species and the overall low prevalence (<10%) of infections observed in adult steelhead returning to the hatcheries despite their overlap with returning adult Chinook salmon, in whom virus prevalence can approach 100%.

Our current study provides further evidence supporting our initial hypothesis that increased levels of stocking of juvenile Chinook salmon in Lake Oroville provided a source and concentrations of virus sufficient to precipitate epidemics at the FRH [23]. The current study also explains a key mechanism by which virus was inadvertently introduced in the hatchery water supply with juvenile Chinook salmon with persistent infections that would not be detected by standard sampling methods that do not include skin. That IHNV was present among Chinook salmon and rainbow trout in Lake Oroville was confirmed by sampling spawning adults captured at the inlet to the lake. Additional support for the hypothesis that the large-scale stocking of Chinook salmon into Lake Oroville was a causative factor for the hatchery epidemics is provided by the rather abrupt and continuing cessation of epidemics following the termination of stocking of juvenile Chinook salmon (from either FRH or IGH) into Lake Oroville.

Bendorf et al. [23] provided experimental evidence that the FRH epidemics could not be traced to the introduction of more virulent strains of IHNV but left unanswered was whether differences in virus susceptibility between populations of Central Valley Chinook salmon or steelhead were potential contributing factors. The current study found almost no evidence of intraspecies variation in IHNV susceptibility within California Chinook salmon, with only one significant difference at a specific temperature. This is similar to findings of Hernandez et al. [25,26], who reported a general lack of intraspecies variation in IHNV susceptibility among Washington and Oregon Chinook salmon, with rare exceptions only in specific cases.

## 5. Conclusions

Based on the absence of variations in fish host susceptibility or viral virulence and the observation of long-term persistence of IHNV in a small proportion of Chinook salmon, we conclude that the amplification of virus in the water source (Lake Oroville) for the hatchery due to stocking high concentrations of Chinook salmon, some of which could have had subclinical/persistent IHNV infections, was the most likely cause of the observed IHNV epidemics at FRH between 1998–2002. In experimental challenge studies Chinook salmon originating within the geographic range of L genogroup IHNV are moderately to highly susceptible to disease and mortality if exposed to the virus as young fry, but susceptibility decreases with age and at the increased temperature of 14 °C. California steelhead are much less susceptible and appear to be a spillover host when reared in close proximity to the more susceptible Chinook salmon. Despite the divergence of L genogroup IHNV into distinct subgroups LI and LII, there is no evidence for differences in virulence, and Chinook salmon populations originating within each subgroup range did not show specific adaptation to the sympatric viral subgroup. Virulence, host specificity and field prevalence [19,23] support the hypothesis that the L genogroup of IHNV has evolved as a specialist virus in Chinook salmon hosts in California.

## Figures and Tables

**Table 1:** California fish populations investigated in virus challenge studies.

Fish Species ^a^	Origin of Population	Geographic Region (Sub-Region)	L Subgroup Range ^b^	History of IHNV Epidemics
Chinook salmon	Feather River Hatchery (FHR)	Sacramento River basin (Feather River)	LII	Yes
Chinook salmon	Nimbus Hatchery (NH)	Sacramento River Basin (American River)	LII	Yes
Chinook salmon	Irongate Hatchery (IGH)	North Coast California (Klamath River)	LI	No
Steelhead trout	Feather River Hatchery (FHR)	Sacramento River Basin (Feather River)	LII	Yes
Steelhead trout	Coleman National Fish Hatchery (CNFH)	Sacramento River Basin (Battle Creek)	LII	No ^c^

^a^ Chinook salmon tested here are all fall-run populations. ^b^ IHNV L subgroup geographic ranges are defined in Kelley et al. [19]. ^c^ Although there have been no IHN epidemics among steelhead trout at CNFH, epidemics among Chinook salmon at the same hatchery occurred regularly in 1941–1999, so the steelhead may have been exposed to virus during that time.

**Table 2:** Isolates of infectious hematopoietic necrosis virus (IHNV) used in experimental challenge studies. Isolates represent five genotypes and three serogroups from the Feather River (FRH) or Coleman National (CNFH) fish hatcheries in California or the Rogue River (RR) in Oregon.

IHNV Isolate	IHNV Isolate Full Name	Origin of Isolate ^a^	Year of Isolation	L Subgroup ^b^	Sequence Type ^b^	Sero-Group
FR04	FR04CA73-13	FRH Chinook salmon Adult	2004	LII	mG011L	3
RR98	RR98CA	RR wild Chinook salmon Adult	1998	LI	mG013L	1
CL02	CL02CA144K1	CNFH Chinook salmon Adult	2002	LII	mG012L	3
FR92	FR92CA61-16	FRH Chinook salmon Adult	1992	LII	mG014L	2
FR69	FR69Clot119	FRH Chinook salmon	1969	LI	mG2107L	1

^a^ All isolates originated from fall-run Chinook salmon, *Oncorhynchus tshawytscha.* ^b^ Phylogenetic subgroups within the L genogroup, species sequence types and serogroups are as described in Kelley et al. [19]. Sequence types have been updated to midG genotypes [10] and correlate with sequence types in [19] as follows: midG genotype mG011L is type u; mG013L is type b; mG012L is type t; mG014 is type n; mG207L is type a.

**Table 3:** Design of experimental studies of Chinook salmon or steelhead trout susceptibility to mortality after exposure to L genogroup IHNV isolates under various conditions.

Exp’t	Fish Species (Populations) ^a^	Life Stage, Ave. Weight	Virus Isolate(s) (L Subgroup) ^b^	Challenge Dose(s) ^c^	Temp(s)
1	Chinook salmon (FRH, NH, IGH)	Fry, 0.54–0.57 g	FR04 (LII), RR98 (LI)	low, intermed., high	12 °C
2	Chinook salmon (FRH, IGH)	Fingerlings, 2.2 g	FR04 (LII)	intermed., high	10, 12, 14 °C
3	Steelhead trout (FRH, CNFH)	Fry, 0.67, 0.68 g	FR04 (LII), CL02 (LII)	low, intermed., high	12 °C

^a^ Chinook salmon populations were from the Feather River (FRH), Nimbus (NH) and Iron Gate Hatcheries (IGH), and steelhead (*O. mykiss*) populations were from the FRH and Coleman National fish hatcheries (CNFH) located in the Central Valley of California. ^b^ Virus isolates are as defined in Table 1. ^c^ Immersion challenge doses of virus were at relative concentrations of 10^4^ (low), 10^5^ (intermediate) or 10^6^ (high) pfu mL^−1^. Actual virus concentrations were determined at challenge and are defined in the methods.

**Table 4:** Detection of IHNV by plaque assay in viral persistence studies in Chinook salmon or steelhead trout. Tissues were collected and processed individually from each fish, and numbers indicate the number of fish positive for virus/number tested.

Fish Species (Populations) ^a^	Life Stage, Ave. Weight at Challenge	Virus Exposure ^a^	120 dpe GKS ^b^	215–216 dpe GKS + Skin	227–248 dpe GKS + Skin
Chinook salmon (NH, IGH)	Fingerlings, 5.1, 4.2 g	FR04 (LII), Low dose	0/60	2/32	Ns ^c^
		Mock	0/60	0/30	ns
Steelhead trout (FRH)	Fry, fingerlings 0.67 g, 1.1 g	FR04, CL02, FR92, FR69 Low/High dose	ns	ns	0/163
		Mock	ns	ns	0/45

^a^ Chinook salmon populations and virus isolates are as defined in Table 1 and Table 2. ^b^ Individual tissues sampled were G, gill; K, kidney; S, spleen, and for later time points, skin. ^c^ ns indicates not sampled at that time point.

## Data Availability

The data that support the findings of this study are openly available from the authors and in the doctoral thesis of C.M.B., University of California, Davis.
